# Targeting residual inflammatory risk in coronary disease: to catch a monkey by its tail

**DOI:** 10.1007/s12471-021-01605-3

**Published:** 2021-08-17

**Authors:** A. T. L. Fiolet, T. S. J. Opstal, M. J. M. Silvis, J. H. Cornel, A. Mosterd

**Affiliations:** 1 Department of Cardiology, University Medical Centre Utrecht, Utrecht, The Netherlands; 2 Department of Cardiology, Meander Medical Centre, Amersfoort, The Netherlands; 3 Netherlands Heart Institute, Utrecht, The Netherlands; 4 The Dutch Network for Cardiovascular Research (WCN), Utrecht, The Netherlands; 5 Department of Cardiology, Northwest Clinics, Alkmaar, The Netherlands; 6 Department of Cardiology, Radboud University Medical Centre, Nijmegen, The Netherlands

**Keywords:** Atherosclerosis, Acute coronary syndrome, Chronic coronary disease, Inflammation

## Abstract

Patients with coronary disease remain at high risk for future cardiovascular events, even with optimal risk factor modification, lipid-lowering drugs and antithrombotic regimens. A myriad of inflammatory pathways contribute to progression of the atherosclerotic burden in these patients. Only in the last few years has the inflammatory biology of atherosclerosis translated into clinical therapeutic options. Low-dose colchicine can provide a clinically relevant reduction in the risk for composite and individual major cardiovascular outcomes in patients with acute and chronic coronary syndromes. Among others, its anti-inflammatory effects in atherosclerosis seem to be related to neutrophil recruitment and adhesion, inflammasome inhibition, and morphological changes in platelets and platelet aggregation. Future research is aimed at further elucidating its particular mechanism of action, as well as identifying patients with the highest expected benefit and evaluating efficacy in other vascular beds. These data will help to formulate the role of colchicine and other anti-inflammatory drugs in patients with coronary disease and atherosclerosis in general in the near future.

## Introduction

Atherosclerosis is a slowly progressive disease. By focusing on lifestyle changes, management of dyslipidaemia and intensive treatment with antithrombotic and anticoagulant agents atherosclerosis-associated mortality rates were lowered by 25–50% in the last two decades [1–3]. Lipid-lowering drugs have had the most notable role in this process, with an estimated 20% relative risk reduction for a major adverse cardiovascular event following every millimole per litre (mmol/l) decrease in low-density lipoprotein (LDL)-cholesterol [4]. Nevertheless, the atherothrombotic risk for patients with coronary disease remains high. Depending on co-morbidities and the extent of the atherosclerotic burden, a given patient will have a 5-year risk of up to 20% for myocardial infarction, ischaemic stroke or death [5, 6].

An inter-individual varying proportion of this risk is explained by the myriad of inflammatory pathways that contribute to progression of the atherosclerotic burden and plaque destabilisation, often referred to as the residual inflammatory risk [7]. Only in the last few years has the inflammatory biology of atherosclerosis translated into several clinical therapeutic options that we will discuss below.

## The magnitude of inflammation as residual risk

Contemporary studies on LDL-cholesterol have provided insight into the magnitude of the remaining risk after optimal treatment of dyslipidaemia. In the *Further Cardiovascular Outcomes Research with PCSK9 Inhibition in Subjects with Elevated Risk* (FOURIER) trial, intensive lipid lowering was attained with statins and the proprotein convertase subtilisin/kexin type 9‑inhibiting molecular antibody evolocumab. Patients achieved a median LDL-cholesterol of 0.78 mmol/l. Even with this state-of-the-art lipid-lowering strategy, 9.8% of patients developed a major adverse cardiovascular event after a median follow-up of just over 2 years [8]. The most important independent contributors to such risk are hypertriglyceridaemia, residual thrombotic risk, diabetes-associated morbidity and low-grade inflammation [9]. When assessing the relevance of the last-mentioned factor, high-sensitivity C‑Reactive Protein (hsCRP) proved a robust prognostic risk marker. Patient-level meta-analyses demonstrated that each standard deviation increment in log-normalised hsCRP was associated with a 37% increase in the relative risk for coronary heart disease. This risk is similar to that associated with an increase in systolic blood pressure (35% relative risk increase per standard deviation increment) and twice as high as the risk associated with an increase in total cholesterol (16% relative risk increase per standard deviation increment) [10].

The pleiotropic effects of statins can be used to estimate the proportional contribution of the residual inflammatory risk. In the *Pravastatin or Atorvastatin Evaluation and Infection Therapy-Thrombolysis in Myocardial Infarction 22* (PROVE IT-TIMI 22) trial and in *The Improved Reduction of Outcomes: Vytorin Efficacy International Trial* (IMPROVE-IT), approximately one third of patients had an hsCRP equal to or above 2 mg/l after achieving an LDL-cholesterol below 1.8 mmol/l. Depending on treatment intensity, statin treatment could reduce median hsCRP by a third, with a greater hsCRP reduction associated with a greater relative risk reduction for cardiovascular events [11, 12]. In addition, the risk reduction following the lowering of hsCRP levels in these patients occurred irrespective of the change in LDL-cholesterol, emphasising the independence of the inflammatory pathway [13].

Mendelian randomisation studies later proved that hsCRP was not causally related to cardiovascular disease [14]. HsCRP, however, is a downstream derivate of the interleukin‑1 and interleukin‑6 inflammatory cascade, which does have a causal role in atherothrombosis [15, 16].

The above-mentioned findings shaped the ‘inflammation hypothesis’ for atherosclerotic disease. Subsequently, multiple both broad-acting as well as targeted agents were introduced to investigate whether anti-inflammatory treatment would truly translate into beneficial effects for patients with atherosclerosis.

## Efficacy of anti-inflammatory drugs in atherosclerosis and coronary disease

### Anti-inflammatory agents with unfavourable effects on cardiovascular events

Non-steroidal anti-inflammatory drugs (NSAIDs) are widely used anti-inflammatory drugs. Their anti-inflammatory properties are not suitable for addressing inflammation in atherosclerosis, as they are consistently associated with an increased risk for major coronary events, with the exception of low-dose acetylsalicylic acid [17]. This is a consequence of the dose-dependent pharmacodynamic properties of the drug. Acetylsalicylic acid inhibits cyclooxygenase (COX)-1 and COX‑2. COX‑1 inhibition reduces thromboxane A2-induced platelet aggregation at low doses, accounting for its atherothrombotic protective effects. At higher doses, the anti-inflammatory properties driven by COX‑2 inhibition arise [18]. Many novel NSAIDs are designed to selectively inhibit only COX‑2. COX‑2 inhibition also leads to undesired cardiovascular effects, such as prostaglandin E2-mediated sodium and water retention, vascular endothelium prostacyclin-mediated platelet activation and vasoconstriction, increasing the risk for cardiovascular events [19].

Glucocorticoids play an integral role in the management of multiple inflammatory conditions. However, many unfavourable side effects such as hypertension, impaired glucose tolerance and obesity following long-term treatment with corticosteroids render them unsuitable for dampening the inflammatory component of atherosclerosis [20].

### Anti-inflammatory agents without effects on cardiovascular events

One of the first attempts to specifically target inflammation in coronary disease was by inhibition of lipoprotein-associated phospholipase A2 (Lp-PLA_2_). Lp-PLA_2_ is bound to LDL-cholesterol and plays a role in oxidative modification within the vascular wall. It increases vascular inflammation in atherosclerosis. The Lp-PLA_2_ inhibitor darapladib was tested in two major trials in almost 20,000 patients with chronic coronary disease and recent acute coronary syndrome. Neither trial demonstrated a reduction in the risk for major cardiovascular events [21, 22]. A variant of the compound inhibiting the secretory form of PLA_2_, varespladib, was associated with a higher rate of recurrent myocardial infarction [23].

Parallel to these efforts, inhibition of the broad p38 mitogen-activated protein (MAP) kinases system was proposed. The p38 MAP kinase system participates in various intracellular signalling routes and is active in endothelial cells, smooth muscle cells and leucocytes. The p38 MAP kinase system inhibitor losmapimod, however, did not show any clinical effect [24].

Methotrexate is a broad-acting immunomodulating drug that inhibits DNA synthesis by competing with folate synthesis and by inhibiting T‑cell adhesion molecules and T‑cell activity. The drug is used in a wide array of auto-immune and oncological conditions. The ability of methotrexate to dampen atherosclerotic inflammation was studied in over 4500 patients with chronic coronary disease and type 2 diabetes or metabolic syndrome. The trial was ceased prematurely for reasons of futility, being unable to demonstrate any sign of clinical benefit [25].

One of the common denominators in these trials was the absence of any evident biochemical response to the treatment. These data strengthened the hypothesis that effective inhibition of the inflammatory pathways in atherosclerosis should comprise targeted and detectable interleukin‑1 and interleukin‑6 inhibition.

### Anti-inflammatory agents with beneficial effects on cardiovascular events

To date, two anti-inflammatory compounds have shown to be efficacious in reducing the risk for major cardiovascular outcomes in patients with coronary disease. *The Canakinumab Antiinflammatory Thrombosis Outcome Study* (CANTOS) was the first to prove that modulating the inflammatory pathway in atherosclerosis reduces major adverse cardiovascular events in patients with a recent history of myocardial infarction and hsCRP equal to or above 2 mg/l. The CANTOS trial randomised 10,061 patients to three subcutaneous doses of canakinumab, the selective interleukin-1β inhibitor, or placebo. Canakinumab 150 mg reduced the risk for the composite end point of non-fatal myocardial infarction, non-fatal stroke or cardiovascular death by 15% and for the composite end point of non-fatal myocardial infarction, non-fatal stroke, cardiovascular death or urgent hospitalisation for angina leading to urgent revascularisation by 17% [16]. Although these results were ground breaking, canakinumab is a costly drug and the findings of the trial have not led to registration of the drug for secondary prevention in cardiovascular disease.

Shortly after CANTOS had demonstrated that selective cytokine inhibition is effective after recent myocardial infarction, two major clinical trials reported on the efficacy of the broad-acting anti-inflammatory drug colchicine in both acute and chronic coronary disease.

*The Colchicine Cardiovascular Outcomes Trial* (COLCOT) randomised 4745 patients within 30 days of myocardial infarction to colchicine 0.5 mg or placebo once daily. The trial showed a 23% relative risk reduction for the composite end point of death from cardiovascular causes, resuscitated cardiac arrest, myocardial infarction, stroke or urgent hospitalisation for angina leading to coronary revascularisation as compared to placebo [26]. *The Low-dose Colchicine 2* (LoDoCo2) trial randomised 5522 patients with chronic coronary disease to 0.5 mg colchicine or placebo once daily and demonstrated a 31% relative risk reduction for the composite end point of cardiovascular death, myocardial infarction, ischaemic stroke or ischaemia-driven revascularisation as compared to placebo [27].

In COLCOT as well as in the LoDoCo2 trial, treatment benefit appeared soon after initiation and continued to accrue over time. Both trials recruited participants treated with optimal medical therapy and irrespective of inflammatory status. The trials excluded patients with severe heart or renal failure, since colchicine is partially eliminated via renal excretion [28]. Exploratory analyses revealed no interaction of treatment with relevant clinical subgroups or concomitant drugs. In the 30-day open-label run-in period used in the LoDoCo2 trial, 9.3% of patients were intolerant to the drug, most often due to benign gastro-intestinal upset. Per the pragmatic design of the trials, major safety parameters were only investigated in an explorative manner. An increased occurrence of pneumonia, seen both with canakinumab in CANTOS and with colchicine in COLCOT, was not observed in the LoDoCo2 trial. The majority of patients in the trials were treated with intensive lipid-lowering agents and many with high-dose statins. No increased occurrence of severe myotoxicity was observed.

## Mechanism of action of colchicine

Colchicine, originally extracted from the autumn crocus (*Colchicum autumnale*), is a widely available drug. It is used chronically to prevent gout and treat familial Mediterranean fever and intermittently for the treatment of pericarditis [29, 30].

Low-dose colchicine is associated with a 30–40% reduction in median hsCRP levels and a 16% reduction in median interleukin‑6 levels in patients with chronic coronary disease [31, 32]. These effects occur early after treatment initiation and are less pronounced than the anti-inflammatory response seen with canakinumab following myocardial infarction [16].

**Fig. 1:**
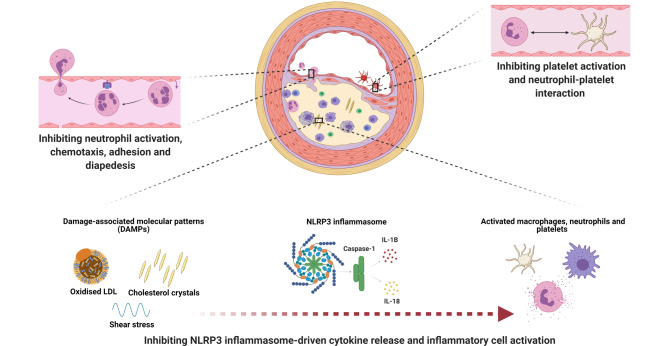
Three different mechanisms that may contribute to the atheroprotective effects of colchicine in atherosclerosis are highlighted. *IL* interleukin, *LDL* low-density lipoprotein, *NLRP3* nucleotide-binding oligomerisation domain-, leucine-rich repeat-, and pyrin domain-containing protein 3

The mechanism of action of colchicine in atherosclerosis relates to the inhibition of microtubule self-assembly. Microtubules are structural components found in various static and dynamic processes of the cell. They form the cytoskeleton, and contribute to the shape and movement of cells. They are also the structures that are used for moving components within the cell. This facilitates cell division (mitosis), intracellular trafficking and secretion of cytokine and chemokines, as well as regulation of ion channels [28]. Microtubules are thus essential in cell movement and activation, the principal part of any inflammatory response. Among others, the anti-inflammatory effects of colchicine are related to the following established concepts in the pathogenesis of atherosclerosis: (1) neutrophil recruitment and adhesion, (2) inflammasome inhibition and (3) morphological changes in platelets and platelet aggregation (Fig. 1).

### Neutrophil chemotaxis, activation and adhesion

Atherosclerotic plaque inflammation is driven by pathological stimuli such as oxidised LDL-cholesterol, blood-pressure-related shear stress and reduced bioavailability of nitric oxide caused by tobacco smoking. The inflammatory response cascades an increasing influx of inflammatory cells such as neutrophils and monocytes that contribute to development of a thin capped fibroatheroma. Structural disturbances of these fibroatheromatas, classically occurring at the cap and shoulders, may lead to erosion or rupture, precipitating atherothrombotic events [33].

Colchicine accumulates with a three- to four-fold higher concentration in neutrophils as compared to mononuclear leucocytes [34]. It reduces the mobility and deformability of neutrophils, thereby inhibiting cell movement and endothelial extravasation. In addition, endothelial adhesion is reduced by means of L‑selectin shedding [35, 36]. Protein expression related to neutrophil degranulation is attenuated by colchicine in patients with coronary disease [32]. The decreased neutrophil mobility and adhesion limits neutrophils entering the plaque. Computed tomography analyses accordingly show fewer high-risk features in patients treated with colchicine than in controls [37].

### Inflammasome inhibition

The microtubule inhibitory properties of colchicine specifically affect inflammasome activity, localised in many immune cells, and macrophages in particular. Activation of the nucleotide-binding oligomerisation domain-, leucine-rich repeat-, and pyrin domain-containing protein 3 (NLRP3) inflammasome leads to caspase‑1 activation and subsequent interleukin-1β and interleukin-18 expression [38]. These cytokines have a flywheel effect in the inflammatory response and increase cell activation and recruitment [39]. Crystalloid structures, among others, contribute to NLRP3 inflammasome activation. The role of this crystal-induced inflammation in gouty arthritis is a result of monosodium urate crystals [38]. In the atherosclerotic plaque, crystallisation of cholesterol can initiate the NLRP3 inflammasome activation and interleukin-1β expression [40]. The attenuating effects of colchicine on NLRP3 inflammasome activity and interleukin-1β may explain its clinical effects in atherosclerosis.

### Platelet morphological changes and aggregation

Microtubules support the shape of non-activated platelets and contribute to the internal reorganisation for shape change after activation. Microtubules and filaments are also structural parts of the pseudopodia of the platelet, used for motility and aggregation. Historical in vitro evidence shows that moderate doses of colchicine impair internal transformation and late aggregation of platelets. High-dose colchicine leads to complete microtubule dissolution and fully halts platelet aggregation [41]. More recently, colchicine was shown to reduce maximal platelet aggregation even after prior COX‑1 and P2Y12 adenosine diphosphate receptor inhibition with acetylsalicylic acid and clopidogrel [42]. Leucocyte-platelet aggregation, rather than platelet-platelet aggregation, seems to be preferentially affected [43]. In vivo evidence of such mechanisms is not yet available. There are no clinical studies that have specially addressed haemostasis or bleeding events associated with colchicine.

## Clinical implementation, unresolved issues and future perspectives

**Fig. 2:**
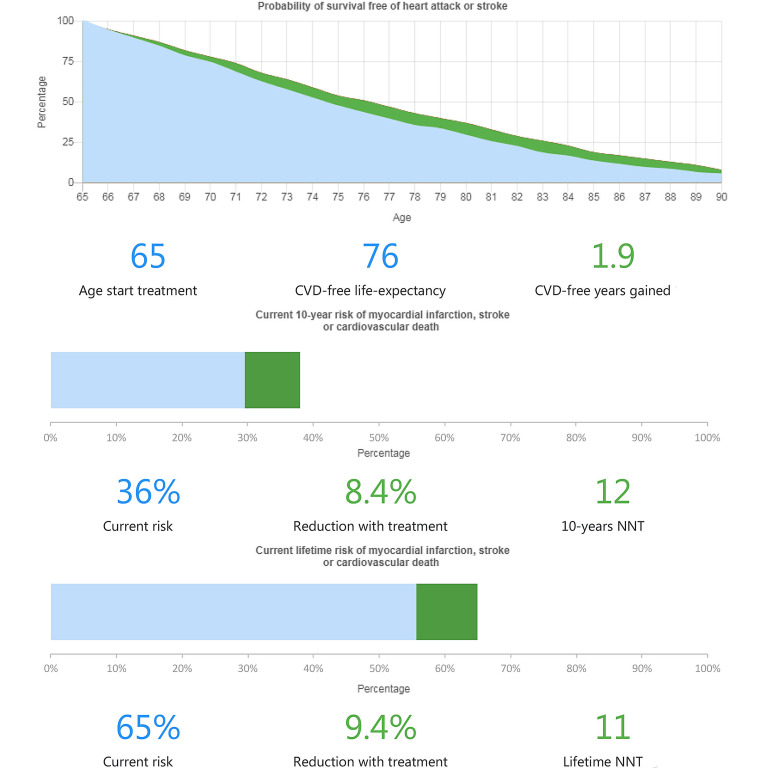
The expected absolute risk reduction and number needed to treat (*NNT*) when colchicine is added to optimal medical treatment. For risk prediction, an out-patient clinic example of a cigarette smoking 65-year old male with coronary disease was selected. He is treated with acetylsalicylic acid, has a systolic blood pressure below 140 mm Hg on antihypertensives and an LDL-cholesterol of 1.8 mmol/l using statins. The *upper panel* shows his probability (in per cent) of being free of myocardial infarction or stroke. The *blue area* under the curve represents his lifetime risk on the current therapy. The *green area* under the curve shows the change in expected lifetime risk after the introduction of anti-inflammatory treatment with colchicine. The *middle panel* shows his 10-year risk for myocardial infarction, stroke or cardiovascular death. The *green bar* shows the absolute reduction in his risk. The *lower panel* shows his lifetime risk for myocardial infarction, stroke or cardiovascular death. The *green bar* shows the absolute reduction in his risk. *CVD* cardiovascular disease, *NNT* number needed to treat

Now multiple large cardiovascular outcome trials have unequivocally demonstrated the clinical efficacy of modulating inflammatory pathways in coronary disease, solid proof of principle of the inflammatory hypothesis in atherosclerotic disease has been provided. When pooled, the results of the low-dose colchicine studies show a relative risk reduction of 25% (relative risk 0.75, 95% confidence interval 0.61–0.91) for major cardiovascular events, with consistent effects on the individual components myocardial infarction, ischaemia-driven coronary revascularisation and stroke [44]. This additional risk reduction, achieved in patients on optimal medical therapy, is a clinically relevant effect that is equal to the risk reduction that can be reached with intensive lipid-lowering therapy and antithrombotic therapy [4, 45]. When trying to translate such effect sizes into daily practice, consider a typical out-patient clinic example of a cigarette smoking 65-year-old man with coronary disease. If treated with acetylsalicylic acid, anti-hypertensive drugs lowering his systolic blood pressure to below 140 mm Hg, and lipid-lowering drugs to achieve an LDL-cholesterol of 1.8 mmol/l, he still has a 10-year risk of 36% for a major cardiovascular event and his lifetime risk will approach 65%. The addition of anti-inflammatory treatment with low-dose colchicine could provide an 8.4% absolute risk reduction to his 10 -year risk for a cardiovascular event and a 9.4% absolute risk reduction for his lifetime risk. This translates into almost 2 years free of major cardiovascular events. (Fig. 2; [46]).

We are only at the beginning of translating the findings from trials on anti-inflammatory therapy into clinical practice. When evaluating the role of such drugs as adjuvant in coronary disease, several questions remain to be answered, among which are the following:

1. How can we identify patients that will benefit most from anti-inflammatory therapy?
2. Will long-term anti-inflammatory treatment translate into a reduction in all-cause mortality?
3. Do patients with atherosclerotic disease of other vascular beds benefit equally?
4. What is the role of alternative highly targeted anti-inflammatory drugs acting on similar pathways?

First, when identifying the ‘right’ patient, the Reynolds risk score and the Secondary Manifestations of ARTerial disease (SMART) risk score demonstrated additional prognostic value of using hsCRP for risk stratification, but have not yet led to clinical recommendation in European guidelines [47, 48]. Clinical risk calculators such as U‑Prevent.com may contribute to estimating the benefit for individual patients based on clinical parameters. The clinical evidence for colchicine was accrued in an ‘all-comer’ population of coronary disease without pre-selection based on inflammatory biomarkers. Multiple ongoing clinical studies are focusing on particular subpopulations (Tab. 1): The ongoing *Colchicine Cardiovascular Outcomes in Acute Coronary Syndrome* trial (COLCARDIO-ACS; ACTRN12616000400460) aims to recruit patients with recent acute coronary syndrome with increased hsCRP levels and hereby determine whether baseline inflammatory status modifies treatment effect. Results are expected in 2025. A substudy of the *Colchicine and Spironolactone in Patients with Myocardial Infarction/SYNERGY Stent Registry* (CLEAR SYNERGY; NCT03048825 and NCT03874338) aims to assess the effect of colchicine on neutrophil activation in response to ST-elevated myocardial infarction, and thus to identify clinical and genetic factors contributing to treatment response.

**Table 1:** Ongoing and planned clinical outcome trials of colchicine in atherosclerosis

Principle investigator	Acronym; Trial Registration	Study name	Patients	Population	Regimen	Maximal follow-up	Primary end point	Estimated completion date
Javed N	No acronym; NCT04218786	Effect of Colchicine in Patients with Myocardial Infarction	800	Myocardial infarction in previous 30 days	Colchicine 0.5 mg once daily vs placebo	3 months	Composite of cardiovascular death, non-fatal myocardial infarction, resuscitated cardiac arrest or hospitalisation for unstable angina	Mid-2021
Kelly P	CONVINCE; NCT02898610	Colchicine for Prevention of Vascular Inflammation in Non-cardio Embolic Stroke	2623	Prior ischaemic stroke or transient ischaemic attack	Colchicine 0.5 mg once daily vs usual care	5 years	Any recurrence of non-fatal ischaemic stroke, non-fatal hospitalisation for unstable angina, myocardial infarction, cardiac arrest or vascular death	End of 2021
Patel S	COLCARDIO-ACS; ACTRN12616000400460	Colchicine Cardiovascular Outcomes in Acute Coronary Syndrome	3000	Acute coronary syndrome in previous 30–45 days and high-sensitivity C‑reactive protein ≥ 2 mg/l	Colchicine 0.5 mg once daily vs placebo	3 years	Composite of cardiovascular death, acute coronary syndrome, urgent revascularisation or non-fatal stroke	2025
Jolly S	CLEAR SYNERGY; NCT03048825	Colchicine and Spironolactone in Patients with Myocardial Infarction/SYNERGY Stent Registry	7000	Acute myocardial infarction with percutaneous coronary revascularisation	Colchicine 0.5 mg once daily vs spironolactone 25 mg once daily vs colchicine 0.5 mg and spironolactone 25 mg vs placebo	3 years	Composite of death, recurrent target vessel myocardial infarction, stroke or ischaemia-driven target vessel revascularisation	2025

Second, when evaluating the net clinical efficacy of the drug, it should be noted that although the combined colchicine trials recruited over 10,000 patients, all-cause mortality in the trials was low. The majority of deaths were non-cardiovascular and the decreased number of cardiovascular deaths was counterbalanced by an increase in non-cardiovascular deaths. The low incidence of fatalities and varying study durations restrict interpretation of these findings. If an intervention delays cardiovascular death substantially, other causes of death may gain the upper hand. Additional studies and long-term follow-up studies will give way to methodological approaches such as competing risk analyses to evaluate long-term effects and evaluate the overall benefit of the drug [49].

Third, when considering other vascular beds, particular interest is given to peripheral artery disease and cerebrovascular disease. None of the trials have yet reported on the incidence or progression of peripheral artery disease. Patients with symptomatic peripheral artery disease are at high risk for adverse cardiovascular events and still have an impressive 10% annual risk for death after revascularisation [50]. Based on the common pathophysiological grounds of coronary disease and peripheral artery disease, a particular protective effect is expected in this population. When considering the effect in cerebrovascular disease, the pooled relative risk reduction of 46% for the risk of strokes was a notable finding in the colchicine trials [44]. *The Colchicine for Prevention of Vascular Inflammation in Non-cardio Embolic Stroke* (CONVINCE; NCT02898610) trial is designed to investigate the effect of colchicine 0.5 mg once daily on the risk for major adverse cerebrovascular and cardiac events in patients with prior cerebrovascular disease in particular. The trial is estimated to complete recruitment at the end of 2021.

Lastly, drugs targeting similar pathways with different pharmacokinetic properties may become increasingly relevant. The interleukin‑1 receptor antagonist anakinra was never able to progress to phase 3 studies in coronary disease. However, the novel interleukin-6 ligand monoclonal antibody ziltivekimab seems effective at reducing multiple inflammatory and thrombotic biomarkers relevant to atherosclerosis. These data have led to the initiation of a large-scale cardiovascular outcomes trial of ziltivekimab compared with placebo in patients with chronic kidney disease who have increased hsCRP and established cardiovascular disease [51]. Selective NLRP3 inflammasome inhibitors are being developed, but no clinical studies have been registered yet.

## Conclusion

In conclusion, in this era of optimal risk factor modification, intensive lipid-lowering and antithrombotic therapy, patients with coronary disease remain at a significant risk for major cardiovascular events. This residual risk partially emanates from the inflammatory drivers of atherosclerosis. The anti-inflammatory drug colchicine can provide a clinically relevant reduction for the risk of major cardiovascular events. Many of its mechanisms of action as well as its efficacy in other atherosclerotic conditions remain to be demonstrated in the near future.
